# Pressure-Aware Operando X‑ray Methods Reveal True Mechanistic Pathways in Solid-State Batteries

**DOI:** 10.1021/acsenergylett.5c03296

**Published:** 2026-01-12

**Authors:** Hung Quoc Nguyen, Juraj Todt, Dragos Stoian, Kenneth Marshall, Elvia Anabela Chavez Panduro, Francois Fihman, Norbert Schell, Günther J. Redhammer, Jozef Keckes, Wouter van Beek, Daniel Rettenwander

**Affiliations:** † Department of Materials Science and Engineering, 8018 NTNU Norwegian University of Science and Technology, 7034 Trondheim, Norway; ‡ Chair of Materials Physics, Montanuniversität Leoben and Erich Schmid Institute for Materials Science, 27268 Austrian Academy of Sciences, 8700 Leoben, Austria; § Swiss-Norwegian Beamlines, 55553 European Synchrotron Radiation Facility, 71 Ave. des Martyrs, 38000 Grenoble, France; ∥ 6Tec, 21 rue du pré des Cieux, 38140 Izeaux, France; ⊥ 28338 Helmholtz-Zentrum Hereon, Max-Planck-Straße 1, 21502 Geesthacht, Germany; # Department of Chemistry and Physics of Materials, 27257 University of Salzburg, 5020 Salzburg, Austria; ∇ Christian Doppler Laboratory for Solid State Batteries, Norwegian University of Science and Technology, 7034 Trondheim, Norway; ○ AIT Austrian Institute of Technology GmbH, 1210 Vienna, Austria; ◆ Institute of Chemical Technologies and Analytics, Vienna University of Technology, 1060 Vienna, Austria

## Abstract

Operando studies of solid-state batteries (SSBs) must capture device-relevant stack pressure and temperature, since uncontrolled conditions can cause relaxation artifacts and lead to false mechanistic interpretations. To address this, we developed an operando framework for X-ray diffraction (XRD) and X-ray spectroscopy (XAS) with precisely controlled dynamic pressure and temperature, deployable across three platforms: (i) scanning microbeam transmission XRD for spatiotemporal mapping of reaction fronts, state-of-charge gradients, and stress localizations; (ii) coupled transmission XRD–XAS for simultaneous tracking of structural and redox evolution; and (iii) laboratory XRD for real-time monitoring of phase transformations during operation. Validated on sulfide-electrolyte SSBs with Li–In anodes and LiNi_0.8_Mn_0.1_Co_0.1_O_2_ (NMC811) or LiCoO_2_ (LCO) cathodes, the framework yields consistent high-quality datasets, which reveal cross-sectional lattice-parameter evolution, spatiotemporal changes in stress gradients, and alteration of structural and redox pathways. By enabling pressure-aware operando XRD and XAS characterization, this framework provides a transferable platform and methodology to minimize artifactual interpretations, ensure reproducible benchmarking, and accelerate mechanistic discovery in next-generation solid-state batteries.

## Graphical abstract

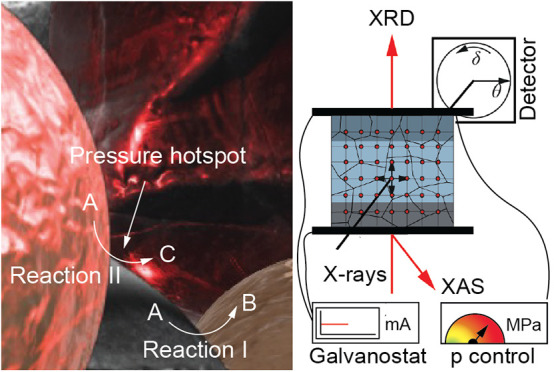

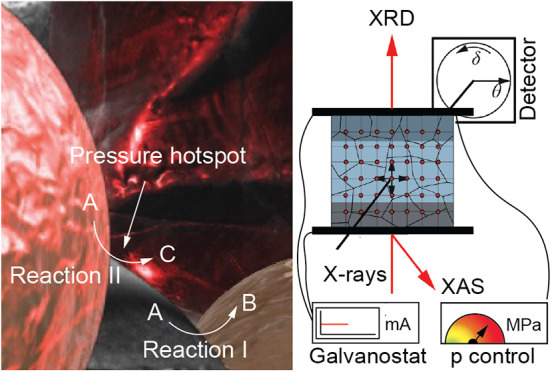

Solid-state batteries (SSBs) are widely regarded as next-generation energy storage, offering higher energy density and improved safety by replacing flammable liquid electrolytes with inorganic solids.^1–3^ Despite this promise, their commercial implementation remains constrained by structural, chemical, and morphological alterations at solid–solid interfaces during operation. Volume changes of active materials and the formation of interphase progressively disrupt the contact area, thereby increasing interfacial resistance and ionic transport tortuosity.^4–8^ Overcoming these limitations requires not only advances in material and interface design but also strategies to determine and potentially adjust the mechanical stresses that arise during operation.

A defining operational variable in SSBs is stack pressure. Continuous contact during cycling generally demands tens to hundreds of MPa, yet the usable range is narrow and system dependent: insufficient pressure degrades percolation, while excessive pressure fractures brittle cathodes or promotes short circuits with Li metal anodes (elastic modulus ≈ 5 GPa).^8–13^ Moreover, the applied macroscopic force does not distribute uniformly at the microscale. Limited particle–particle contacts and structural heterogeneity concentrate stresses into local “hot spots” that can approach the gigapascal regime.^4,14,15^ Processing steps such as calendering further densify and texture composite electrodes, altering contact networks, and seeding residual stresses that intensify during cycling.^16–18^ Microstructure-resolved simulations predict stresses up to ∼3 GPa at particle surfaces, directly linking electrode architecture, densification, and stress localization.^19^

Such extremes alter both thermodynamics and kinetics. For example, BiF_3_ cathodes in fluoride-ion systems display a nonmonotonic pressure dependence, with ionic transport suppressed at low pressure and parasitic transformations triggered at high pressure.^20^ In anionic-redox layered oxides, pressure modifies lattice parameters and transition metal–oxygen covalency, shifts the onset and hysteresis of oxygen redox, and can drive irreversible cation rearrangements.^21^ Localized stresses of ca. 300 MPa stabilize a metastable Na_2_S_3_ intermediate in Na–S cells,^22^ while high current densities in Li_10_GeP_2_S_12_ electrolytes promote stress-driven β → γ transformations in Li_3_PS_4_, altering ion-transport pathways.^23^ Together, these results demonstrate that pressure and its heterogeneity directly shape reaction mechanisms and phase stability (see also Figure 1).

**Figure 1:**
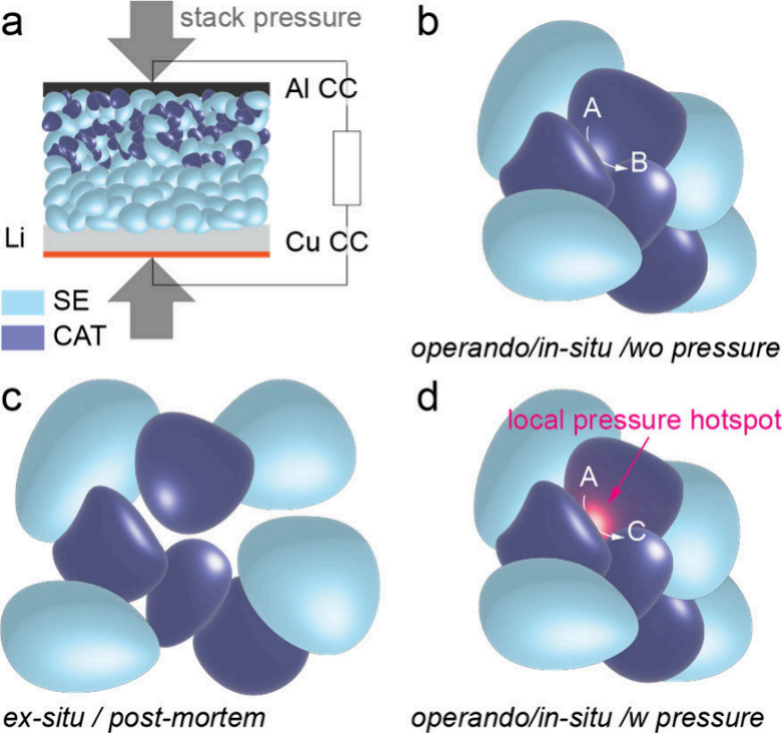
**Operando environment matters.** (a) Schematic of an all-solid-state battery with a Li-metal anode operated under applied stack pressure. (b) Operando/in situ measurements without applied or well-controlled pressure can report apparent reaction pathways dominated by loss of solid–solid contact and transport-limited heterogeneity. (c) Ex situ/post mortem analyses at ambient pressure often yield similar, relaxation-biased interpretations. (d) Under controlled stack pressure during measurement, heterogeneous contact produces local “pressure hot spots” that can reach the GPa regime, thereby altering reaction pathways and interphase formation.

Moreover, chemo-mechanical degradation amplifies state-of-charge (SOC) gradients in composite electrodes.^24,25^ Finite ionic and electronic transport across the cathode produces overpotential gradients through the electrode thickness, such that different regions traverse the (de)­lithiation pathway asynchronously.^26^ Because transport properties themselves depend on SOC, these gradients sharpen dynamically during cycling and are exacerbated when mechanical degradation raises local resistance.^27,28^ The feedback between contact loss, increasing resistance, and nonuniform transport accelerates SOC heterogeneity, particularly under high current or imperfect percolation.^29,30^ Bulk-averaged probes then superimpose signals from zones at different SOC, giving rise to apparent multiphase behavior or broadened transitions that mimic intrinsic phase coexistence.^31,32^

Taken together, pressure hot spots, electrochemistry–pressure coupling, and SOC gradients all generate artifacts that can obscure or distort mechanistic interpretation. Operando studies must therefore replicate the true pressure–temperature (*P*–*T*) window of operation while ideally resolving spatial heterogeneity.^33,34^

To address these challenges, we developed an operando X-ray device that allows for stack-pressure and temperature controlled multimodal characterization. The framework comprises three approaches: (i) scanning microbeam transmission XRD to resolve micrometer-scale reaction fronts, SOC and strain gradients, and stress localization; (ii) coupled XRD–XAS to directly couple lattice evolution with element-specific redox and short-range order; and (iii) a laboratory XRD setup that enables time-resolved, pressure-controlled in-house studies. Together, these methods provide a transferable platform to probe the coupled structural, electronic, and mechanical processes that govern solid-state battery function under realistic operating conditions.

To directly probe the chemo-mechanical landscape of working SSBs, we applied our newly developed operando device which works in two configurations (transmission and cross-section mode) across three applications: for (i) scanning microbeam XRD for resolving reaction fronts, SOC and strain gradients, and stress localization in cross-section mode; for (ii) synchronized XRD–XAS for coupling lattice evolution to element-specific redox conditions in transmission mode; and for (iii) a laboratory diffractometer for time-resolved in-house studies in transmission mode. This section demonstrates how each approach captures structural, electronic, and mechanical processes under device-relevant conditions, beginning with spatially resolved mapping of phase and stress evolution across electrode cross sections.

## Spatiotemporal Phase and Stress Evolution along the SSB Cross Section

Operando X-ray powder diffraction experiments typically provide volume-averaged data, hiding microstructural heterogeneities across electrode layers. During operation, overpotential gradients across the cathode thickness lead to heterogeneous SOC, which evolves dynamically since transport properties depend on SOC itself. Thus, apparent phase coexistence in averaged data may actually reflect SOC gradients. Cross-sectional mapping avoids this artifact by probing structural, chemical, and mechanical changes at micrometer resolution.

We tested this capability with our device, loaded with a solid-state cell comprising an NMC811/LICF composite cathode, a Li_6_PS_5_Cl solid electrolyte, and a Li–In alloy anode. The experiment was performed at PETRA III (DESY, P07-EH1) in transmission geometry (Figures 2a,b, and S2d). A microfocused beam (10 μm vertical; down to 1 μm is possible at P07-EH3) was scanned through the electrode thickness in discrete steps equal to the beam size. The cell was galvanostatically cycled between 2.8 and 4.3 V vs Li^+^/Li at 25 mA g^–1^ under constant stack pressure of 85 MPa (Figure 2c).

**Figure 2:**
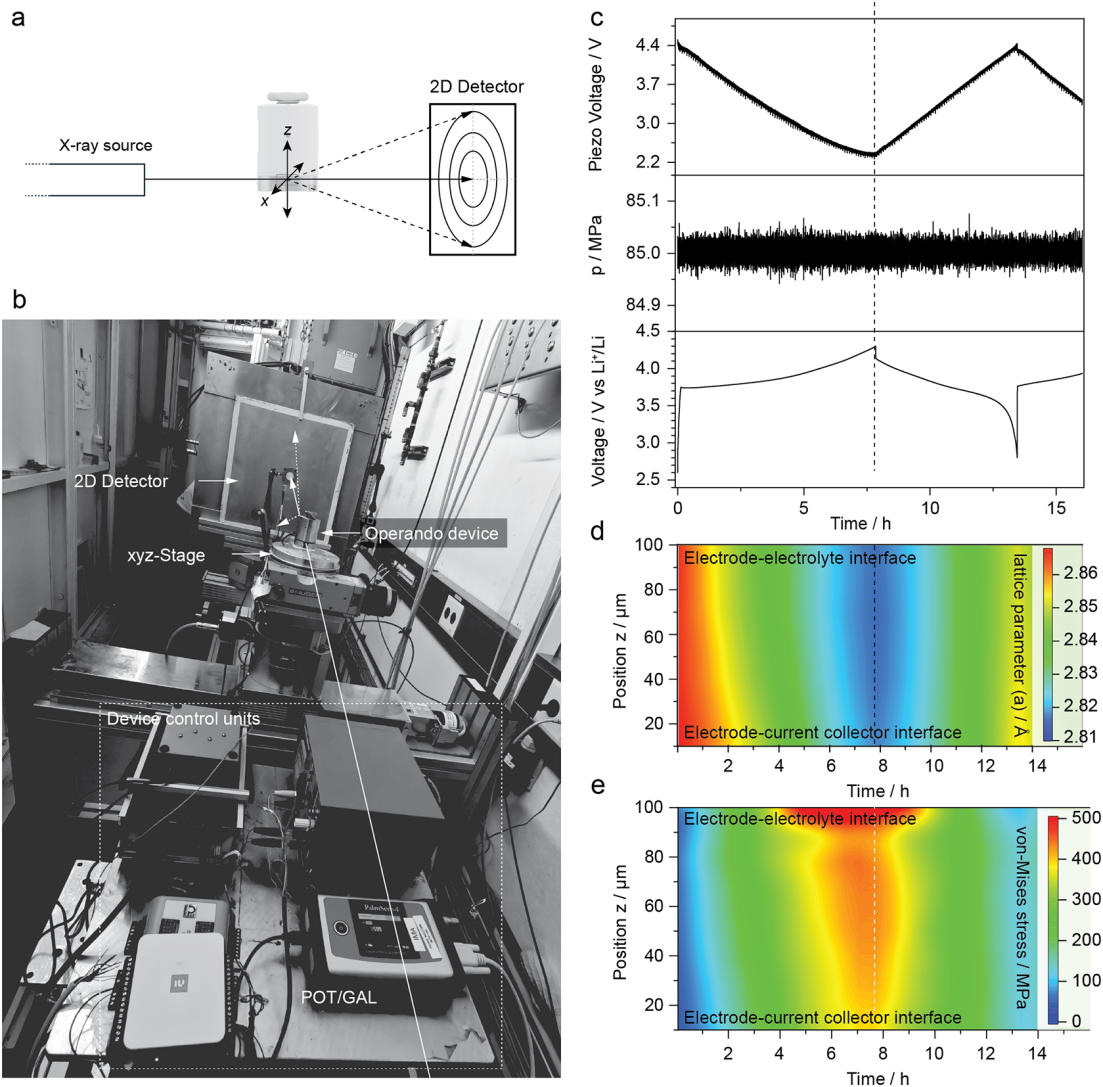
**Operando scanning microbeam X-ray diffraction.** (a) Transmission-geometry schematic of the scanning configuration. (b) Photograph of the operando cell on a motorized *xyz* stage at DESY (PETRA III, P07-EH1), with potentiostat and control electronics. (c–e) Time-resolved experiment on a solid-state battery with Li_6_PS_5_Cl electrolyte, a NMC811/Li_6_PS_5_Cl/C composite positive electrode, and LiIn as negative electrode. (c) Piezo-actuator voltage, stack pressure, and cell voltage, demonstrating stable closed-loop pressure control during cycling. (d) Spatiotemporal map of the NMC811 basal-plane lattice parameter *a* (position = through-thickness coordinate *z*), extracted from 2D diffraction. (e) Corresponding effective von Mises stress map across the stack, revealing stress localization that evolves with state of charge.

Electrochemical data showed that the cathode delivered a first charge capacity of 196 mAh g^–1^ and a discharge capacity of 140 mAh g^–1^, corresponding to a first-cycle efficiency of 71.4%. This relatively low efficiency is consistent with sluggish Li kinetics at high Li contents in Ni-rich layered oxides^35,36^ as well as with interfacial degradation between high-voltage cathodes and sulfide electrolytes.^37,38^ The stack pressure remained constant at 85 MPa throughout cycling, with fluctuations < ±50 kPa, demonstrating reliable closed-loop control. The piezo actuator voltage decreased during charge (compensating for expansion) and increased during discharge, consistent with electrode breathing. While delithiation of NMC811 usually contracts the lattice,^39^ the overall cell expanded, showing that the Li–In alloy anode dominated volume changes. The limited 15 μm actuator stroke allowed only relative displacement monitoring, but calibration would enable the extraction of absolute dilatometric data. Finally, we note that the same device can operate in a constant-volume mode by disabling dynamic pressure control such that stack pressure evolves naturally with state of charge. Results from constant-volume experiments are provided in Figure S9.

Cross-sectional mapping of the NMC811 lattice parameter *a* (Figure 2d) revealed pronounced anisotropic and asymmetric reaction fronts. During delithiation, a sharp front emerged at the cathode–electrolyte interface and propagated toward the current collector, producing a steep lattice gradient across the electrode thickness. Lithiation reversed this progression, but the front was significantly broader and more diffuse, indicating slower Li reinsertion and incomplete reversal of the delithiation pathway. This asymmetry highlights kinetic limitations in lithiation, likely governed by a combination of sluggish interfacial charge transfer and bulk Li diffusion.

Simultaneous stress analysis (Figure 2e) showed von-Mises equivalent stresses up to 435 MPa in the bulk cathode and exceeding 500 MPa near the separator at full charge. These stresses relaxed reversibly upon discharge, indicating that fracture thresholds were not exceeded. Since stresses observed are averaged along the beam direction, this hints toward pressure hotspots that may exceed by far the GPa regime, potentially allowing for metastable phase formation.

## Tracking Phase and Redox Chemistry Simultaneously

While cross-sectional diffraction captures phase gradients and stress evolution, it does not reveal the associated redox chemistry or how short-range structure and amorphous phases evolve. To overcome this, we combined operando XRD with XAS at ESRF BM31 (SNBL), enabling the simultaneous collection of diffraction patterns and element-specific spectra from the same sample volume (Figures 3a,b and S2c). Typical acquisition times were a few seconds for XRD and some minutes for XAS, providing near-synchronous structural and electronic information under constant stack pressure and temperature. Performing XAS in transmission mode, which is possible for elements with sufficiently high absorption edges, allows for simultaneous tracking of XRD and XAS. Figure 3c summarizes the correlated dataset for the NMC811 cathode during cycling between 2.8 and 4.3 V. In this specific case, the transition-metal valence changes extracted from XAS predominantly reflect the material near the cathode–current-collector interface. This spatial selectivity arises from the presence of heavy elements such as indium (introduced via LICF), whose strong absorption at the relevant low-energy edges severely limits X-ray penetration. As a result, XAS measurements were performed in fluorescence mode rather than transmission mode. Additional details are provided in Figure S10.

**Figure 3:**
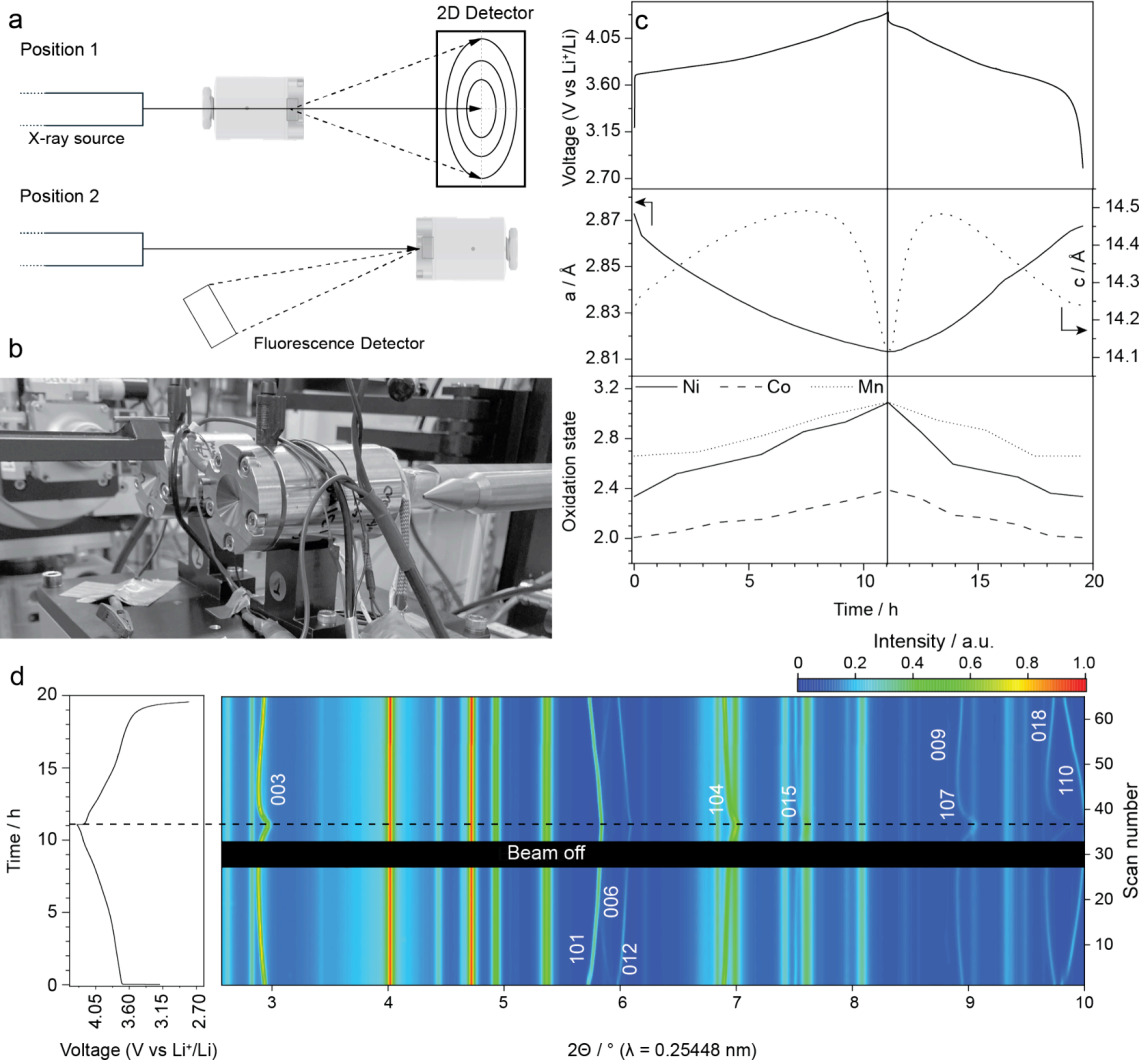
**Simultaneous operando XRD–XAS.** (a) Schematics of the transmission XRD geometry (2D area detector) and fluorescence XAS geometry. (b) Photograph of the setup at SNBL/ESRF (BM31) showing the operando cell mounted on a motorized stage. (c) Correlated structural and electronic evolution of a solid-state battery with Li_6_PS_5_Cl electrolyte, an NMC811/Li_6_PS_5_Cl/C composite positive electrode, and LiIn as negative electrode: lattice parameters *a* (left axis) and *c* (right axis) from Rietveld refinement of diffraction and oxidation states from XANES (Ni, Co, Mn). The nearly monotonic decrease of *a* and the nonmonotonic *c* lattice parameter changes reflect layered-oxide (de)­lithiation, while Ni dominates the redox response; Co and Mn remain comparatively invariant. The XAS spectra of Ni, Mn, and Co during battery cycling are shown in Figure S7. (d) The right plot presents the time–2θ intensity map of operando diffraction patterns highlighting indexed NMC811 reflections (e.g., 003, 101, 006, 012, 104, 015, 107, 018, 110); the “beam-off” band marks short interruptions. The left plot shows the corresponding electrochemical charge/discharge of the battery cell. All data were collected under constant, closed-loop stack pressure.

The *a* lattice parameter contracted nearly linearly from 2.8718 to 2.8152 Å, coinciding with a progressive increase of the Ni oxidation state from ca. +2.4 to +3.1, while Co and Mn remained comparatively stable at ca. + 2.2 and ca. +2.8, respectively. These trends demonstrate that Ni dominates charge compensation. In contrast, the *c*-parameter displayed a nonmonotonic response: expansion from 14.2136 to 14.4912 Å during charging up to ca. 4.1 V, followed by a sharp collapse as the upper cutoff was approached. On discharge, both *a* and *c* retraced their paths, indicating structural reversibility (Figure 3c,d). The initial *c*-axis expansion arises from the reduced ionic radius of oxidized Ni and enhanced Ni–O covalency, whereas the collapse reflects destabilization of the layered framework upon deep delithiation.^39^

By synchronizing XRD and XAS, we directly couple lattice strain to specific redox processes, demonstrating that Ni oxidation drives basal-plane contraction while interlayer collapse originates from high-voltage lattice destabilization. Such mechanistic assignment would not be possible from either technique alone, and uncontrolled-pressure measurements would potentially blur these trends due to contact loss or structural relaxation for some electrode materials.

This approach is particularly valuable for conversion-type electrodes such as FeF_2_, MnO_2_, SnO_2_, MoSe_2_, CuS, and Se which undergo multielectron redox reactions frequently accompanied by amorphization, nanoscale reorganization, and formation of transient intermediates. These processes are often invisible to diffraction because long-range order collapses early in cycling. XAS, however, can follow oxidation states and changes in local coordination environment throughout the reaction pathway, while XRD can capture any crystalline intermediates that form transiently. For instance, FeF_2_ has been shown to convert through intermediate Fe–F phases before yielding metallic Fe and LiF,^40^ while MnO_2_ proceeds via multiple redox steps involving spinel-like intermediates.^41^ The exact pathway can vary depending on electrolyte chemistry, which may stabilize or suppress or even form alternative specific intermediates. Moreover, the large volume changes (>100%) characteristic of conversion electrodes necessitate controlled stack pressure to maintain interfacial contact and mitigate electro-chemo-mechanical degradation. High overpotentials associated with conversion reactions also require elevated temperatures. The simultaneous application of XRD and XAS in a dedicated pressure- and temperature-controlled device uniquely enables mechanistic resolution under such realistic operating conditions.

Taken together, synchronized XRD–XAS under controlled pressure and temperature provides a powerful framework for disentangling structural–electronic coupling, capturing metastable intermediates, and identifying pressure- and electrolyte-dependent pathways. These capabilities extend well beyond layered oxides, offering new opportunities to resolve the reaction mechanism in solid-state batteries, considering both thermodynamic variables, pressure and temperature.

## Laboratory Diffractometer: Time-Resolved Phase Evolution

Although synchrotron XRD provides high brilliance, resolution, and fast acquisition, access is limited by beamtime constraints and high competition. In contrast, laboratory diffractometers are broadly available at universities and research institutes, allowing continuous operation and flexible scheduling. Adapting our operando device for in-house diffractometers thus enables routine mechanistic studies under realistic solid-state battery conditions. To demonstrate this capability, we conducted operando experiments on a Bruker D8 Advance diffractometer equipped with a Mo anode X-ray source (λ ≈ 0.71 Å) and a focusing mirror optics. The Mo wavelength offers greater penetration through the cell stack compared to conventional Cu radiation (λ ≈ 1.54 Å), making it well suited for transmission geometry. The operando cell was mounted on the XYZ stage of the diffractometer (Figure 4a–c), connected to a portable potentiostat and data acquisition electronics. Alignment was confirmed using a NIST Si standard powder prior to measurements.

**Figure 4:**
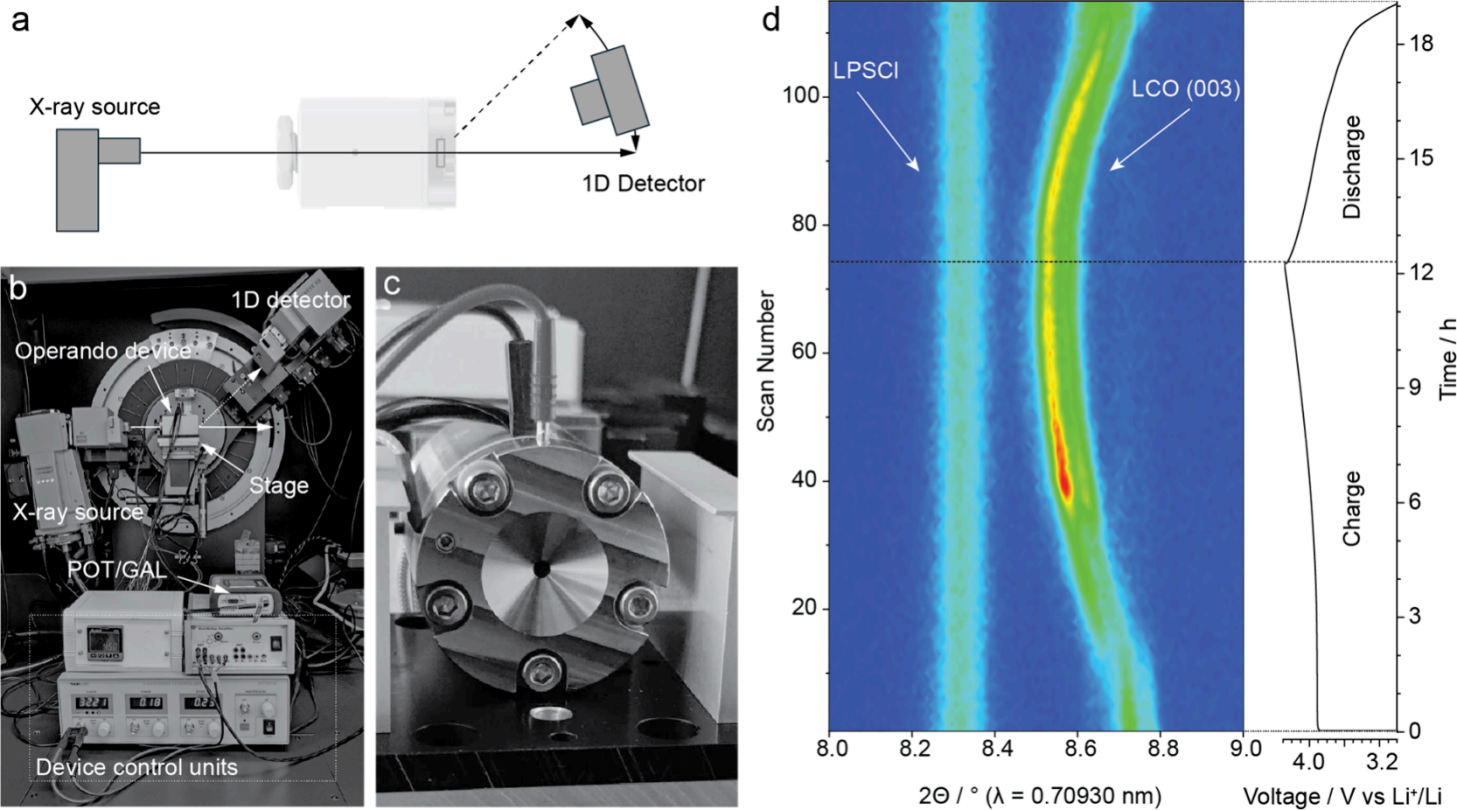
**Operando XRD using lab diffractometer.** (a) Schematic of the operando X-ray diffraction experimental arrangement in transmission geometry, featuring the operando cell for real-time structural characterization using an in-house diffractometer system with a maximum scan range of 2θ from 0° to 45°. (b, c) Photographs of the operando cell mounted on the motorized *xyz* stage of a Bruker D8 Advance diffractometer. Cell positioning was optimized through stage translation until the characteristic XRD pattern of the silicon reference standard was obtained, confirming proper beam alignment. The operando cell was connected to a portable potentiostat and associated control/data acquisition devices; (d) Time-resolved operando XRD patterns (left panel) tracking the 003 reflection of LiCoO_2_ and the 002 reflection of Li_6_PS_5_Cl solid electrolyte, correlated with the electrochemical charge/discharge curve (right panel).

As a model system, we cycled a LiCoO_2_ (LCO) cathode between 3.0–4.3 V vs Li^+^/Li at a current density of 12 mA g^–1^ and a temperature of 45 °C, with a constant stack pressure of 55 MPa applied throughout. To balance time resolution with signal quality, diffraction scans were restricted to 8–9° 2θ, covering the 003 reflection of LCO and the 002 reflection of the Li_6_PS_5_Cl electrolyte. Each scan required ∼6 min, enabling time-resolved monitoring of structural changes during cycling. For completeness, a full-range scan (5–40° 2θ) was also collected (Figure S1). The operando dataset (Figure 4d) clearly resolved shifts of the LCO 003 Bragg reflection during delithiation and lithiation, reflecting the well-known expansion and contraction of the *c* axis in layered oxides. Lithium removal weakens the Coulombic interaction between the CoO_2_ slabs and interlayer Li, causing expansion during charge, while reinsertion restores the interaction and contracts the lattice during discharge.^42–44^ These lattice changes are consistent with the expected electrochemical breathing of LCO. In contrast, the Li_6_PS_5_Cl electrolyte displayed no discernible changes in its diffraction peaks over the same potential window, confirming its structural stability under the applied thermal and pressure conditions.

These results demonstrate that our compact operando device even enables reliable, time-resolved in-house XRD studies under device-relevant stack pressure and elevated temperature. Importantly, the capability to capture mechanistic structural changes on a conventional diffractometer broadens accessibility beyond synchrotron facilities, enabling 24/7 local studies, systematic pre-screening of materials, and reproducibility testing. This portability ensures that operando characterization with controlled pressure and temperature can be implemented widely, thereby accelerating standardized benchmarking of solid-state battery materials and architectures.

Unraveling the true mechanisms in solid-state batteries demands experiments that reproduce their operating environment. Stack pressure and temperature are critical variables: if neglected, relaxation effects and localized pressure hot spots can obscure structural and electrochemical pathways, leading to misleading interpretations. Operando studies must therefore be performed under realistic, closed-loop pressure–temperature control to resolve the genuine coupling between structure, redox, and mechanics.

Here, we have established operando X-ray methodologies that meet this requirement across both synchrotron and laboratory platforms. The approach proves particularly powerful for (i) mapping micrometer-scale reaction fronts, SOC and strain gradients, and stress localization using scanning microbeam XRD; (ii) directly linking lattice evolution with element-specific redox processes via synchronized XRD–XAS; and (iii) enabling time-resolved in-house studies with a Mo-source diffractometer, without the need for bespoke cell geometries.

By making pressure-aware operando characterization both practical and transferable, this work lays the foundation for avoiding mechanistic artifacts, establishing standardized benchmarks, and accelerating the design of next-generation solid-state batteries. Moreover, the capability to systematically vary stack pressure opens new opportunities to probe the pressure dependence of materials within the relevant operating window, deepening our understanding of structural, chemical, and mechanical phase behavior, and ultimately advancing the rational engineering of solid-state battery chemistries and architectures.

## Supplementary Material



## Notes

Materials and Methods, note on the working principle of the operando device, note on tracking phases and structural evolution, note on stress analysis with scanning microbeam X-ray diffraction, refinement data of NMC811 and LPSCl, full-range XRD pattern of the operando in-house XRD, diagram of the working principle of the operando device, DRLAB GUI, illustration of the operando device for operando X-ray diffraction experiments and scanning microbeam X-ray diffraction, typical Rietveld refinement plot of the refined SXRD pattern, and position of the cell components derived from refinement of the X-ray diffraction scan (PDF)
