# Supplementary material for: Pressure-Aware Operando X‑ray Methods Reveal True Mechanistic Pathways in Solid-State Batteries
Source: ACS Energy Lett. 2026 Jan 12;11(2):1761–8. doi: 10.1021/acsenergylett.5c03296 (PMC12910653; doi:10.1021/acsenergylett.5c03296)
Supplement: Supplementary file 1 [file nz5c03296_si_001.pdf]

Supporting Information

Pressure-aware operando X-ray methods reveal true mechanistic pathways in solid-state batteries

Hung Quoc Nguyen[1], Juraj Todt[2], Dragos Stoian[3], Kenneth Marshall[3], Elvia Anabela Chavez Panduro[1], Francois Fihman[4], Norbert Schell[5], Günther J. Redhammer[6], Jozef Keckes[2], Wouter van Beek[3], Daniel Rettenwander[1,7,8,9]*

[1] *Department of Materials Science and Engineering, NTNU Norwegian University of Science and Technology, 7034 Trondheim, Norway*

[2] *Chair of Materials Physics, Montanuniversität Leoben and Erich Schmid Institute for Materials Science, Austrian Academy of Sciences, 8700 Leoben, Austria*

[3] *Swiss-Norwegian Beamlines, European Synchrotron Radiation Facility, 71 Ave. des Martyrs, 38000 Grenoble, France*

[4] *6Tec, 21 rue du pré des Cieux, 38140 Izeaux, France*

[5] *Helmholtz-Zentrum Hereon, Max-Planck-Straße 1, 21502 Geesthacht, Germany*

[6] *Department of Chemistry and Physics of Materials, University of Salzburg, 5020 Salzburg, Austria*

[7] *Christian Doppler Laboratory for Solid State Batteries, Norwegian University of Science and Technology, 7034 Trondheim, Norway*

[8] *AIT Austrian Institute of Technology GmbH, 1210 Vienna, Austria*

[9] *Institute of Chemical Technologies and Analytics, Vienna University of Technology, 1060 Vienna, Austria*

*Daniel.rettenwander@ntnu.no

Materials and Methods

1 Materials preparation

Synthesis of $Li_{0.2}In$ (LI). LI, as the anode, was fabricated by pressing lithium foil (Sigma Aldrich) onto indium foil (Fisher Scientific) using a hydraulic press. Specifically, 1 ton was applied for a period of one hour. The molar ratio of lithium to indium was maintained at 1:5. Following this process, the LI foil was carefully punched into 5 mm discs for subsequent experimental use.

Preparation of cathode composites. Single-crystalline $LiNi_{0.8}Mn_{0.1}Co_{0.1}O_2$ (NMC811, MTI) or $LiCoO_2$ (LCO, Cerpotek AS) was ground with $Li_3InCl_{5.4}F_{0.6}$ (LICF) in a weight ratio of 57:43. This mixture was processed using a planetary ball mill (MSE Supplies). A 45 ml ZrO_2 jar containing 5 mm ZrO_2 balls was employed, with a ball-to-powder ratio set at 30:1. The milling process consisted of 12 cycles, each comprising 10 minutes of milling followed by 10 minutes of rest. All milling operations were conducted under an argon atmosphere to prevent oxidation, utilizing a custom-made holder for the milling jar.

2 Physicochemical characterizations

Design considerations. The operando cell was designed to combine versatility with realistic operating conditions of solid-state batteries. Its compact geometry allows for mounting on synchrotron beamlines (here, BM31 at ESRF and P07 at DESY), as well as on laboratory diffractometers such as a Bruker D8 DaVinci, enabling a seamless transition between large-scale and in-house experiments. To support transmission-mode XRD, X-ray energies above 17 keV (Molybdenum radiation) were targeted to achieve sufficient penetration through both cell windows (0.5 mm in thickness) and the electrochemical stack, with a typical 2θ range of 0–45° selected to capture the strongest reflections of most electrode and electrolyte phases. The design further permits adjustment of angular range when different stack pressures and corresponding window thicknesses are applied, balancing diffraction coverage with mechanical loading. All components were selected to resist degradation under high pressures (up to 200 MPa), elevated temperatures

(up to 150 °C), and contact with reactive alkali metals, while electrical isolation of the stack minimizes external noise during diffraction and spectroscopic measurements. These considerations together yield a robust, modular device architecture capable of multimodal operando characterization under realistic solid-state battery conditions.

In cross-sectional measurements, the X-ray beam transmits through the cell body and only the material layer of interest. The X-ray transparency of the cell housing enables such experiments to be performed conveniently at most synchrotron XRD beamlines. To avoid additional diffraction peaks arising from the partial crystallinity of PEEK,[1] polyetherimide (PEI) was used instead.[2] PEI (Ultem, McMaster-Carr) is fully amorphous, mechanically robust, and significantly less expensive than PEEK or Kapton, while contributing no parasitic diffraction.

For through-plane measurements, where the X-ray beam must penetrate the entire battery stack, a metallic anode can act as an X-ray-blocking layer. To prevent beam obstruction, a ring-shaped Li–In electrode with an ~1 mm opening (Fig. S5) was employed, allowing the beam to pass exclusively through the cathode and electrolyte. The window required for cross-plane measurements must combine high X-ray transparency with electrical conductivity and mechanical stability. Although beryllium offers excellent transmission, safety concerns associated with oxidized Be render it unsuitable.[3] Glassy carbon (HTW Hochtemperatur Werkstoffe GmbH) was therefore selected as the optimal window material. Glassy carbon (HTW Hochtemperatur Werkstoffe GmbH) was therefore selected as the optimal window material. Its bulk density is approximately 1.4–1.5 g%cm^{-3}, and it exhibits low ash content (<100 ppm), which minimizes parasitic absorption and scattering from impurities. Glassy carbon also provides a favorable combination of electrical and mechanical properties for use as a current collector and structural window. Its specific electrical resistance is on the order of 5×10^{-3} Ω cm, ensuring sufficient in-plane conductivity for operando measurements. Mechanically, it has a flexural strength of about 260 N%mm^{-2}, compressive strength of roughly 580 N%mm^{-2}, and a Young's modulus near 35 kN%mm^{-2}, providing adequate stiffness and robustness under stack pressures up to tens of MPa.

Hermetic sealing was maintained using two O-rings, one for each plunger. Electrochemical impedance spectroscopy of the device assembled with densified LPSCl (Fig. S8) shows no significant changes in either the high- or low-frequency regimes over three days, indicating that the cell remains stable outside the glovebox.

The operating principle of the operando device is illustrated in Fig. S2a and discussed in Note S1. Closed-loop control during cycling is fully enabled by the DRLAB software suite, a LabVIEW-based platform with a DAQExpress interface (Fig. S2b).

Operando X-ray diffraction. Powder samples of cell components (NMC811, LPSCl, and LICF) were loaded into 0.3 mm glass capillaries (Hilgenberg GmbH) for pre-characterization and for refining the crystal structures for subsequent multi-component operando measurements. The XRD patterns of these samples were collected in a transmission mode at the Swiss-Norwegian Beamline (SNBL) beamline BM01 at the European Synchrotron Radiation Facility (ESRF, Grenoble, France) using a monochromatic, high-brilliance X-ray beam. The experiments utilized a beam size of 150 × 300 μm^2 with a wavelength of λ = 0.78242 Å. Data were collected using a two-dimensional (2D) detector (2M Pilatus) and rotating the capillary by 300° with a 30-second measuring time. Data processing and integration were done using the Bubble software.[4] The detector position calibration was achieved by measuring a standard silicon reference powder and employing the calibration module provided by the pyFAI software package.[5]

“Quasi-simultaneous" operando synchrotron X-ray diffraction and X-ray absorption near-edge structure (XANES) measurements of the battery cell in the cross-plane direction were performed at the ESRF, SNBL-BM31 beamline.[6] The experiments commenced with XRD measurements in transmission geometry, utilizing a monochromatic, high-brilliance X-ray beam with λ = 0.25448 Å and dimensions of 300 µm horizontally and 50 µm vertically. Data recording was facilitated using a two-dimensional 2M Pilatus detector, and the cell assembly was moved during the 30-second collection time by 1 mm perpendicular to the beam to obtain better counting statistics with the detector positioned 89.114 cm away from the sample. Again, the XRD data processing and integration were done using the Bubble software,[4] and the detector position was calibrated against a silicon reference powder. For the detail analysis procedure, see Note S2.

Subsequent operando XANES measurements were performed on the Mn K-edge (energy range: 6.44 –6.69 keV), Ni K-edge (8.23–8.48 keV), and Co K-edge (7.61–7.86 keV) in

fluorescence mode. The energy was scanned by a double-crystal Si(111) monochromator operated in the continuous scanning mode (~65 seconds per spectrum, four repetitions). For calibration and comparison, known standards were measured in transmission mode prior to the experiment. The chosen standards were an elemental Co-foil, Ni-foil, Mn-foil, as well as NiO, $LiNiO_2$, CoO, Co_3O_4, MnO, MnO_2, and Mn_2O_3 pellets. The Athena software (part of the Demeter package) was used to process the raw data (calibration, normalization, background subtraction) and to extract the XANES signal for further use, plotting, and interpretation.[7]

Scanning micro-beam X-ray diffraction. Diffraction experiments were conducted at the High Energy Materials Science Beamline (HEMS) side-station P07b, operated by Helmholtz Zentrum Hereon at the PETRA III synchrotron of DESY in Hamburg, Germany. A monochromatic X-ray beam with $\lambda = 0.14125$ Å (87.1 keV) and dimensions of 500 μm horizontally and 10 μm vertically was used. The beam was scanned across the entire stack of cell layers with a step size of 10 μm over a range of 150 μm, completing each scan in 5 minutes. This scanning method enabled effective mapping of thickness-position versus discharge/charge time in the cell.

Powder-like diffraction patterns were collected at each mapped point using a Perkin Elmer XRD 1621 Flat Panel area-sensitive X-ray detector with a 2048 x 2048 pixels resolution, positioned 1481 mm downstream from the sample. The detector geometry relative to the gauge volume was calibrated using a NIST LaB6 standard powder, following procedures provided by the pyFAI software package.[5] The detail analysis is shown in Note S3.

3 Electrochemical characterization

Preparation of operando battery cells. The components and construction of the operando cell are detailed in a separate section. This section only shows how to assemble the battery stack. Briefly, 20 mg of Li_6PS_5Cl (LPSCl, sourced from Nei Chemical) was placed into the cell insulation sleeve, which has a center diameter of 5 mm. The LPSCl powder was compacted using a hydraulic press between two steel plungers at a pressure of 380 MPa for 3 minutes. Subsequently, 2.79 mg of the prepared cathode powder was applied to one side of the densified pellet, while a 5 mm LI disc was

placed on the opposite side. The entire battery stack was then compressed at 150 MPa for 3 minutes. Finally, the sleeves containing the battery stack were assembled into the cell setup for subsequent use.

Galvanostatic cycling. Cycling tests were conducted with a total cathode loading of 2.79 mg (14.22 mg/cm²), corresponding to an NMC811 loading of 1.59 mg (8.1 mg/cm²). The cells were maintained at a constant pressure of 85 MPa during battery cycling, using a dynamic pressure-controlling protocol. The applied current for cycling was 31.8 μA (161.2 μA/cm²), equivalent to 20 mA/g. The lower and upper cutoff potentials were set at 2.8 V and 4.3 V versus Li^+/Li, respectively, with the potential of LI being 0.62 V versus Li^+/Li. Galvanostatic charge-discharge cycling was performed using a PalmSens4 potentiostat, and data were recorded with PSTrace 10.0 software.

4 Declaration of Generative AI and AI-assisted Technologies in the Writing Process

The authors used Perplexity, Gemini (Google), and Grammarly AI (Grammarly) to improve the readability, grammar, consistency, and language of this work. After using these tools, the authors reviewed and edited the content as needed, taking full responsibility for the content of the publication.

Supplementary Notes

Note S1. Working principle of the operando device

The working principle of the operando device is illustrated in Fig. S2. During battery cycling, uniaxial force is measured using a Wheatstone bridge-based load cell ZMMC4 (Anhui Zhimin Electrical Technology) with a 10 V excitation voltage. The load cell output signal is fed to a custom amplifier (gain = 100) for signal conditioning and amplification. Real-time voltage acquisition is performed using a data acquisition module NI-USB-6002 (National Instruments), which converts the amplified signal to pressure values (MPa). These pressure measurements are then used to calculate the required control voltage for the piezoelectric actuator PK44M3B8P2 (Thorlabs).

The system employs proportional control, where the output voltage of the data acquisition module (0-10 V) drives a piezoelectric controller MDT694B (Thorlabs) with 0-150 V regulation capability. The controller incrementally adjusts the piezo voltage (Control factor) until the measured pressure converges to the setpoint within a predefined tolerance range (Delta P).

Note S2. Tracking phases and structural evolution

Synchrotron X-ray diffraction data were evaluated using the Rietveld technique as implemented in the program TOPAS V6. In all refinements, the background was modelled using a Chebychev 5th order polynomial function. The amorphous components arising from the electrochemical cell (or the capillary, see below) were modelled using pseudo-Voigt-shaped single peaks. Number, position and shape of these were extracted from measurement on the empty capillaries (for the measurements on the individual cell components) or from the calibration measurement of the empty cell with the silicon standard inside. For the crystalline phases, the peak shape was modelled with a Thompson-Cox-Hastings modified pseudo-Voigt function (PV-TCHZ) with 6 refinable parameters.

For getting good starting models, NMC811, LPSCl and LICF were investigated in detail using sXRD data obtained from capillary measurements with high *q*-values. The as-purchased NMC811 sample is phase pure, showing trigonal $R\overline{3}mH$ (IT No. 166) symmetry. In structure refinement, peak shape and lattice parameters, fractional atomic coordinated (z O1) and the *B*-values of all symmetry non-equivalent atoms were refined to reliable values. For both, the Li1 (3*a*) and the M2 (3*b*) sites, full site occupation was assumed. It is known from literature that Li-rich NMC cathode materials may contain Li^+/Ni^{2+} disorder.[8-13] Thus, for the 3*a* site, the site occupancy was refined as $Li^+ + Ni^{2+} = 1$, allowing for Ni^{2+} anti-site defects, while for the 3*b* site the Co and Mn contents were fixed to the values, provided by the supplier (0.1 apfu respectively), while a migration of Li onto the 3b was allowed by applying the constraint $Ni^{3+} + Li^+ = 0.8$, following a procedure applied e.g. by Orlova et al.[5] This gives a small amount of Ni – anti-site defects of 0.02 apfu on 3*a*, while vice-versa almost the same amount of Li^+ migrates to the 3*b* site, maintaining overall Ni content to 0.8 apfu (see Table S1 for details). The atomic displacement parameters B were refined isotropically and were constrained to be the same for all elements on the same non-equivalent sites, i.e. Li + Ni on the 3*a* site have the same B – value, the same accounts for the cations on the 3*b* (TM) site, but they were allowed to refine freely for the distinct site. Results of the structure refinement are given in Tables S1.

For LPSCl, the structural data of Schlenker,[14] ICSD#133976 were used as starting parameters for refinement of the synchrotron XRD data of the pure component, allowing the lattice parameter, the atomic coordinates (sulphur atom S1) and the isotropic atomic displacement parameters of all non – Li atoms to refine freely. The Li - positions were fixed on the values found in literature from neutron diffraction experiment, while the site occupancy was allowed to refine. Results of these refinements are given in Table S2. For LICF, structural data of Helm et al. 2021,[15] ICSD#122395 were used as starting parameters for refinement against the experimental synchrotron XRD data of this cell-component.

Evaluation of XRD data of the complete cell-assemblage of combined XRD/ XAS operando cycling experiments was done in a sequential mode making use of the so-called lauch-mode in TOPAS. Due to their complexity and multiphase-nature of the XRD pattern in the operando measurements, the peak shape parameters of individual phases were fixed to the values obtained from the capillary measurements of individual components. To account for any possible changes in peak width, a Lorentzian (crystallite size) broadening was allowed in sequential refinements. However, there was no significant change in peak shape and width within the resolution of the present measurements. This means that obviously there is no detectable change in crystallite size during cycling. The structural parameters (atomic coordinates, B-values, site occupation numbers) of NMC811 were constrained with only the lattice parameters, the scaling factors of the cell components and the site occupation factor of Li1 ($3a$) site being allowed to vary freely. For LPSCl and LICF, lattice parameters, scaling factors and an individual crystallite size broadening parameter was allowed to refine. A typical Rietveld refinement plot of the refined SXRD pattern of the initial cell is shown in Fig. S3. The phases present are NMC811 with 9.3(4) wt %, LICF with 36.2(8) wt. %, LPSCl with 45.2 wt.% and finally iron from the cell with 9.3 wt %. Broad bumps in the data were modelled by individual lines and arise from the glassy carbon cell windows.

A very similar refinement strategy as described above was also applied to the data collected from PETRA III. Due to the spatial resolution of the beam, it is possible to scan the different cell components along the z – axis of the cell. By this, it is possible to get good estimates of the thicknesses of different components and also to do some pre-characterization of the material. Before starting with the detailed scans, the cell was completely scanned giving rise to the different regions of the cell, starting with mainly taenite in the beginning with NMC811, LPSCl and LICF

increasing towards 50 μm, then decreasing and vanishing above 100 μm. Due to the thickness of the solid-state electrolyte, LPSC is dominating between 100 and 500 μm (see Fig. S4), while toward above the anode material (Li/In foil and taenite from the plungers) become the main phases. The maximum amount of NMC811 reaches ~ 35 wt% which is sufficient high to extract detailed structural behaviour upon electrochemical treatment. The electrochemical testing was the done in the region between 0 and 100 μm in a finer slicing along z. In these screening before cycling the lattice parameters of NMC remained constant within the estimated standard deviations.

Note S3. Stress analysis with scanning micro-beam X-ray diffraction

Powder-like 2D diffraction patterns were collected while repeatedly scanning across the battery stack layers, resulting in position- and time-resolved maps. Each diffractogram was integrated azimuthally into 36 10°-wide sections, further reflecting the orientation-dependence of the diffraction signal. In each of these sections, peaks corresponding to the {003}, {015}, {018}, {101}, {104}, {110} and {113} $R\overline{3}m$ NMC lattice place families were fitted with pseudo-Voigt peak functions to determine their orientation-dependent Bragg angle, from which then orientation-dependent lattice plane spacings were derived. Since no strain-free reference was measured during the experiment, the averages with respect to orientation and position from the first scan were employed as reference values. Since these values are fixed, but the NMC lattice parameters change in dependence on the SOC, a virtual volumetric (hydrostatic) stress arises during standard multi-axial stress analysis as described for instance by Meindlhumer et al.[16] There are two ways to address this, either by (i) subtracting a volumetric stress calculated from the lattice parameter evolution evaluated by Rietveld analysis on the same diffraction data and literature data for the bulk modulus of NMC, or (ii) by considering only the difference between the axial and tangential/radial stress component, which is not affected by this volumetric part. In any case, a well-suited measure for interpreting stresses obtained from dissimilar experiments is the von Mises stress, as it gives effective absolute (unsigned) values that can be compared with yield strength and ultimate strength values obtained from other analytical techniques or literature. Besides the mentioned normal stress differences, the von Mises approach also considers shear stresses that also remain broadly unaffected by any volumetric component. Therefore, the von Mises metric was chosen with this study, calculated directly from the axial to tangential/radial stress difference and shear stress components. Finally, since XRD analysis directly provides only elastic strains, single-crystal elastic constants (i.e. the stiffness tensor) calculated by Liu et al.[17] also had to be employed in conjunction with a Hill grain interaction model to relate strains to stresses.

Supplementary Tables

Table S1. Rietveld analysis results of NMC811 from synchrotron XRD diffraction data, λ = 0.632430 Å, trigonal symmetry, $R\bar{3}mH$, a = 2.87332(8) Å. c = 14.20028(6) Å.

Atom	Site	x	y	z	B	Occ
Li1	3*a*	0	0	0	2.07(14)	0.979(2)
Ni1	3*a*	0	0	0	2.07(14)	0.021(2)
Ni2	3*b*	0	0	½	1.70(2))	0.778(3)
Li2	3*b*	0	0	½	1.70	0.022(3)
Mn2	3*b*	0	0	½	1.70(2)	0.1*
Co2	3*b*	0	0	½	1.70(2)	0.1*
O	6*c*	0	0	0.24100(12)	1.81(3)	1.00*

* value fixed during refinement, site occupation restraints: Li1 + Ni1 = 1, Li2 + Ni2 = 0.8

Table S2. Rietveld analysis results of LPSCl from synchrotron XRD diffraction data, λ = 0.632430 Å, cubic symmetry, $F\bar{4}3m$, a = 9.75793(5) Å.

Atom	x	y	z	B	Occ
Li1	0.3229	0.0257	0.6771	3	0.3403
Li2	0.047	0.254	0.547	3	0.1
P	0.5	0.5	0.5	1.518	1
S1	0.12056	-0.12056	0.62056	2.937	1
Cl	0	0	0	3.151	1
S2	0.75	0.75	0.75	1.517	1

Supplementary Figures

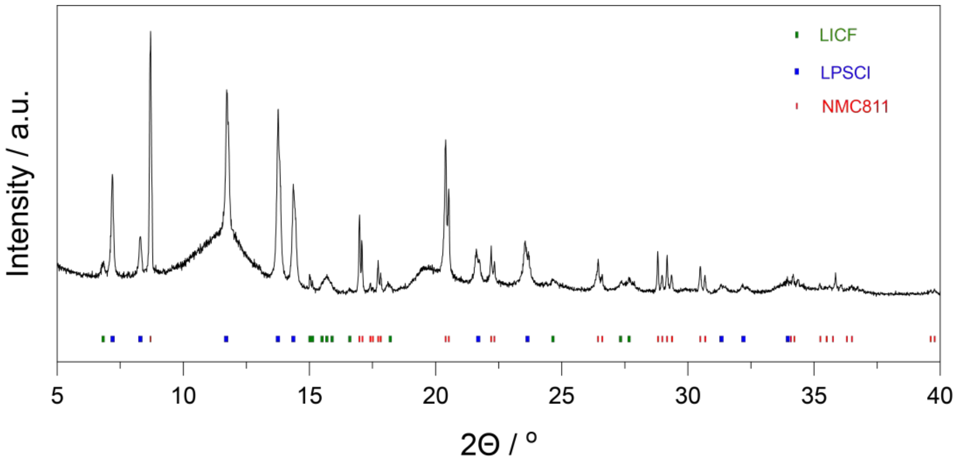

Figure S1. Full range XRD pattern of the operando in-house XRD battery taken with Mo-radiation from 5-40 degrees. The wide background bumps around 11 and 20 degrees are related to the amorphous nature of glassy carbon windows of the cell. Each scan took one hour to have a decent quality of data. Therefore, in the operando experiment, the scan is focused on the 8-9 2θ which required approximately 6 minutes for each scanning cycle.

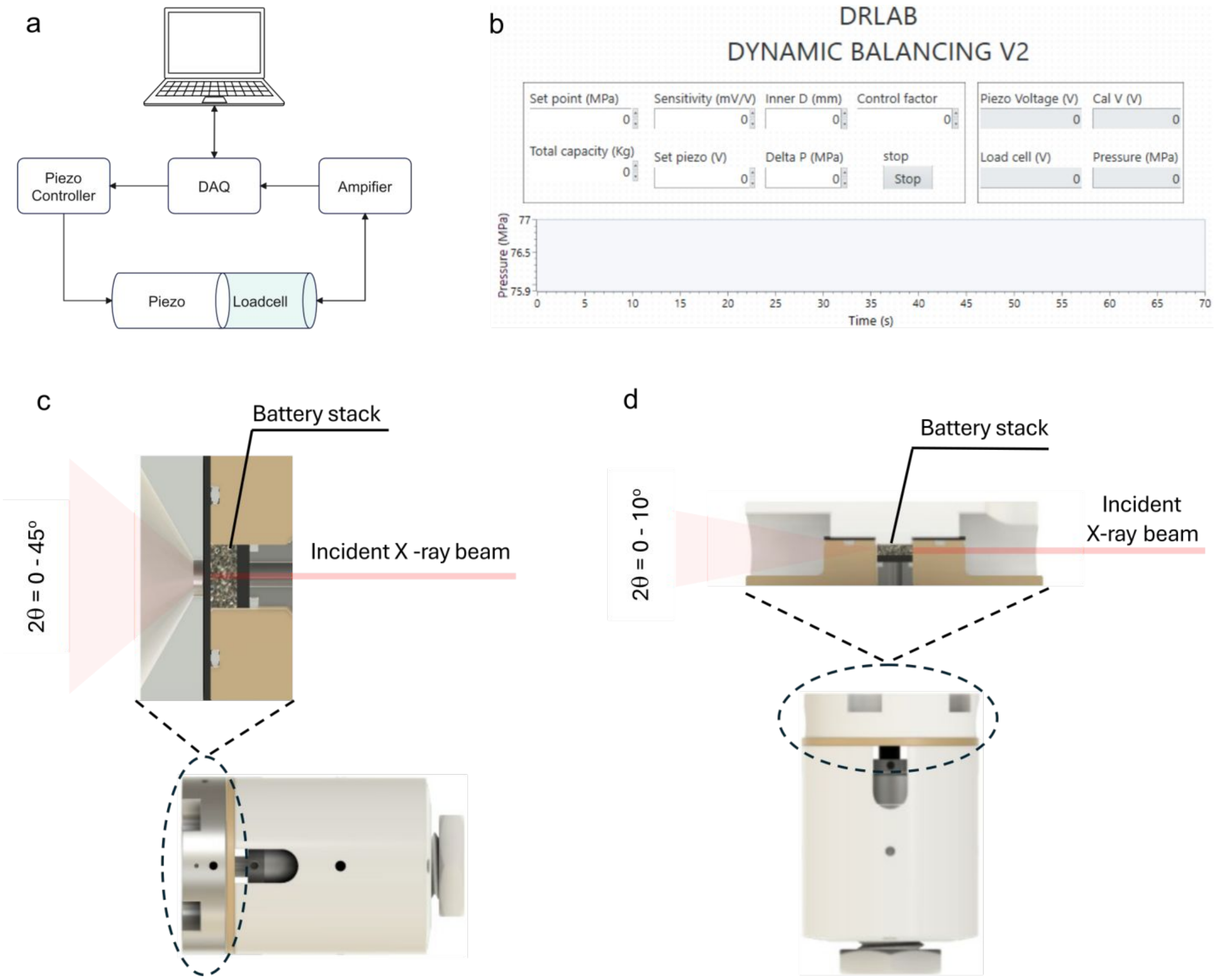

Figure S2: (a) The working principle of the operando device. (b) The DRLAB software GUI for dynamically controlling and displaying the pressure of the operando device. (c) The configuration of the operando device for operando X-ray diffraction experiments. (d) The configuration of the operando device for scanning micro-beam X-ray diffraction.

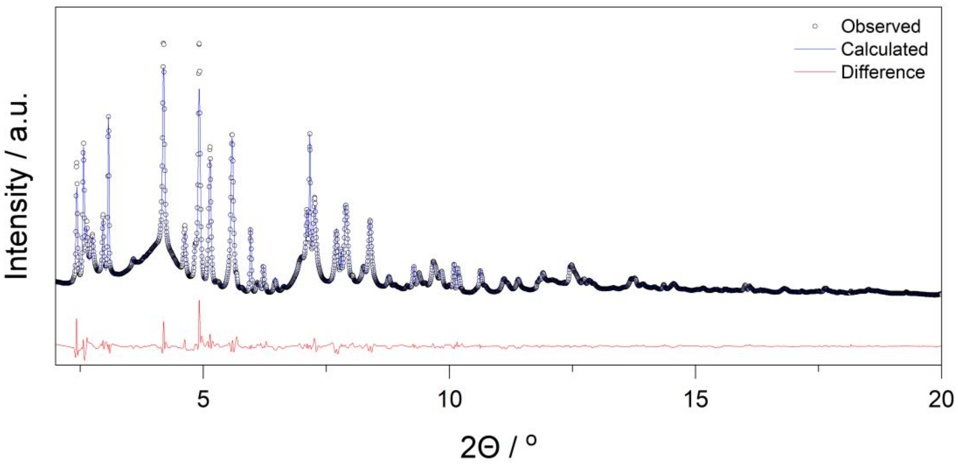

Figure S3: A typical Rietveld refinement plot of the refined SXRD pattern (λ = 0.25448 Å) of the initial cell for the simultaneous operando XRD and XAS measurement.

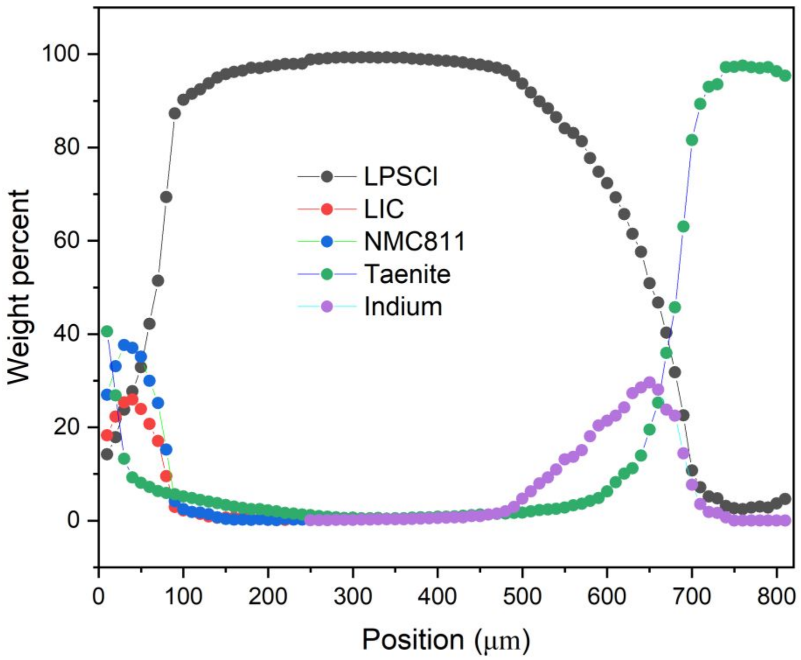

Figure S4: Position of the cell components derived from refinement of the X-ray diffraction scan through 800 μm thickness of the cell. As a result, the detailed scans only focused on the region with the highest concentration of NMC811 (10–100 μm).

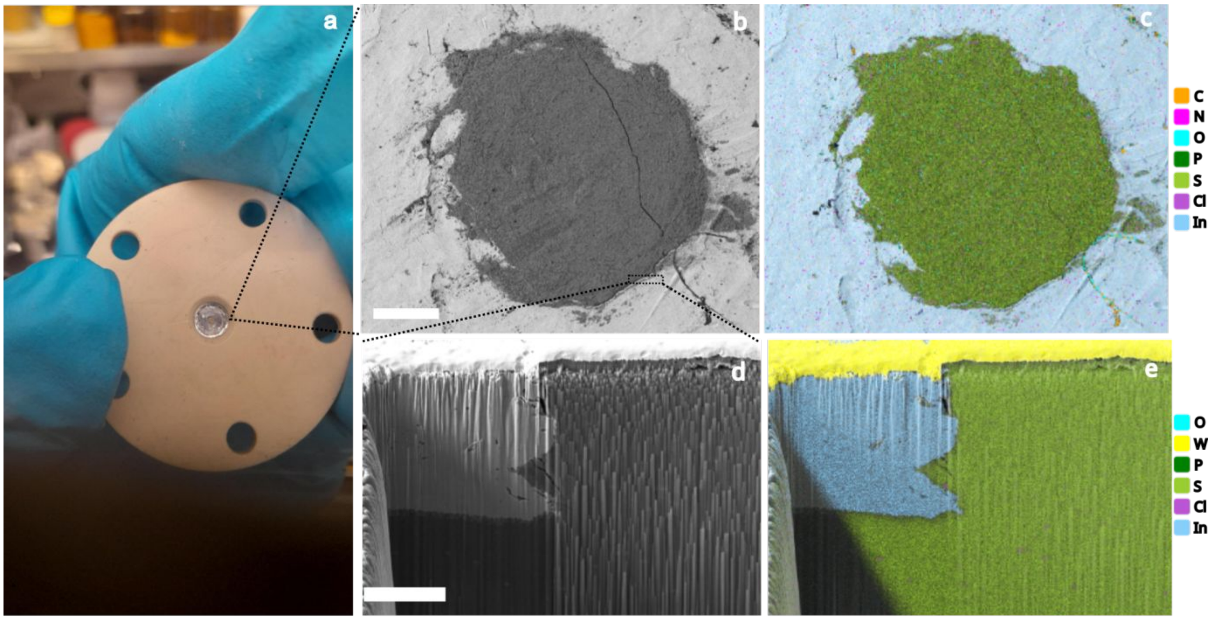

Figure S5. The anode side of the battery stack. (a) Picture of the back side of the battery stack where the anode is a ring with outer diameter of 5 mm and the inner diameter is 1 mm. The hole in the center of the anode is useful to avoid blocking of the X-ray. (b) and (c) SEM image and EDX mapping focus on the center area of the anode side. The LiIn anode and the LPSCl electrolyte is clearly distinguished. (d) and (e) FIB-SEM image and corresponding EDX mapping of the small edge area highlighted in (b). The tungsten layer is used only for protecting the surface of the electrode being milled. The scale bars in (b) and (d) are 20 μm.

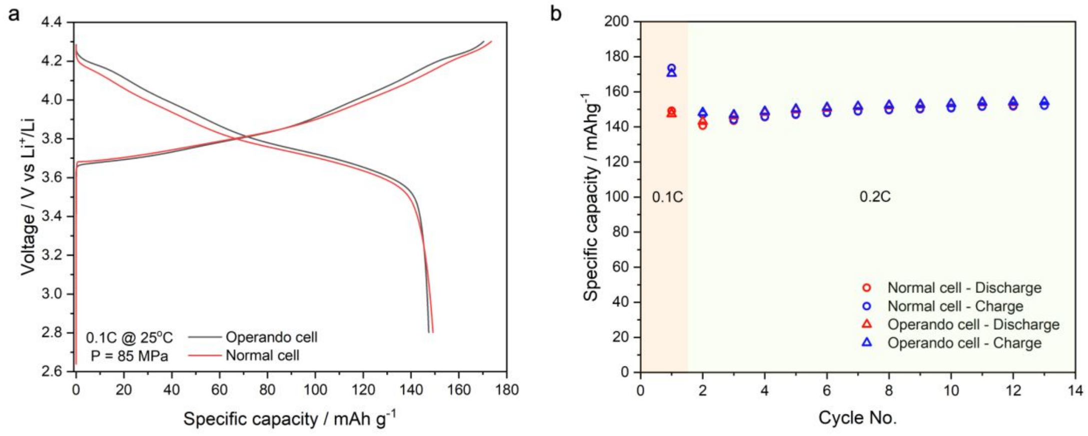

Figure S6. Cycling performance of the Operando device. (a) Charge/discharge curve comparison of the first cycle between the normal cell using in the lab and the operando device. Both cells achieved almost similar capacities from the charge/discharge curves and the number of plateaus. The small variation in the overpotential between the two cells may be attributed to the variation of the anode during preparation. (b) Cycling performance of the same battery cells under current densities of 0.2C. The cycling performance of the two cells presented in Figure S6b shows similar behavior. The capacity of both cells dropped slightly after applying higher current density is a common phenomenon. The capacities were then recovered in the first few cycles before being stable from the 8th cycle, which may indicate that there was a need for some time to activate the active material of the cell.

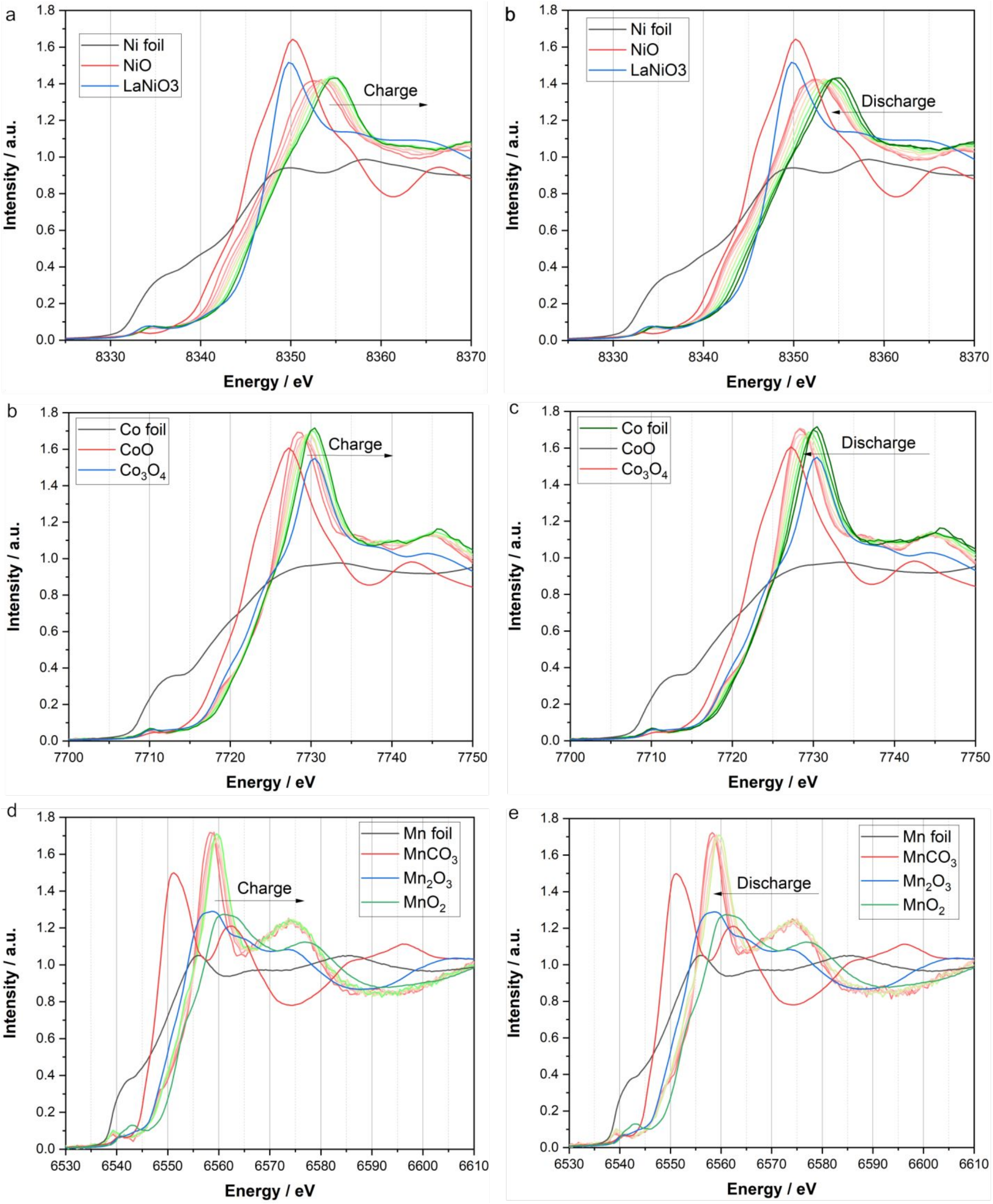

Figure S7: XAS spectrum during charge/discharge of the electrode material with corresponding reference materials. (a) and (b) Ni K-edge, (c) and (d) Co K-edge, (d) and (e) Mn K-edge.

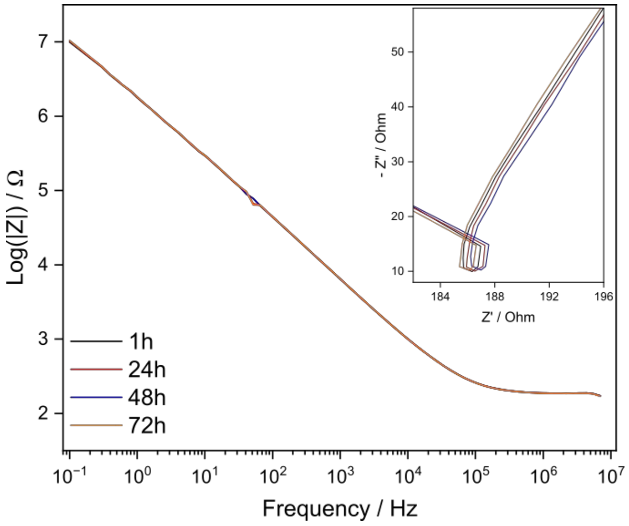

Figure S8: EIS of the device with densified LPSCl. The main graph is the Bode plot of the EIS spectrum conducted from 7 MHz to 100 mHz after placing the device outside of the glovebox for 1 hour, 24 hours, 48 hours, and 72 hours. There is no obvious change in the spectrum which indicates that the device is stable when working outside of the glovebox for long time. The inset graph is the Nyquist plot of the spectrum at high frequencies domain. There are only minor variations in the resistance of the electrolyte due to the minor change of the temperature of the testing lab.

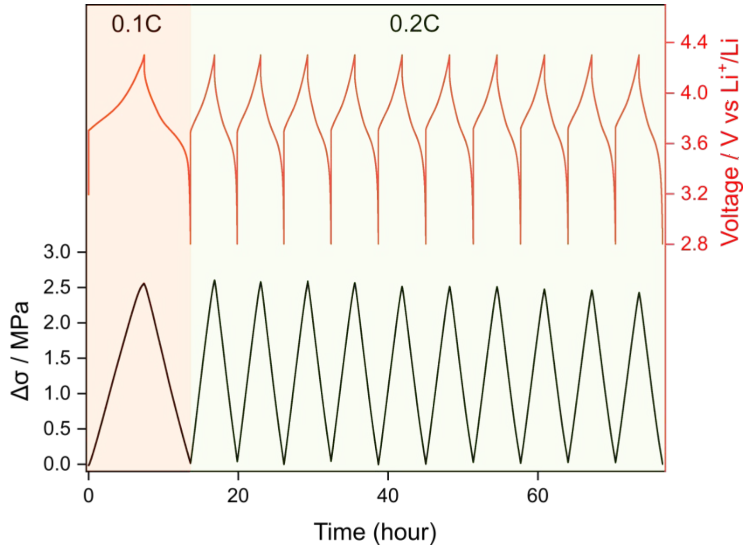

Figure S9: Constant volume mode of the operando device. The plot shows the cycling of a battery stack using NMC811 cathode, LPSCl electrolyte, and LiIn anode without controlling pressure of the device. The initial stack pressure is set at 85 MPa and is recorded during the battery cycling. The pressure of the device naturally evolved with an amplitude of approximately 2.5 MPa.

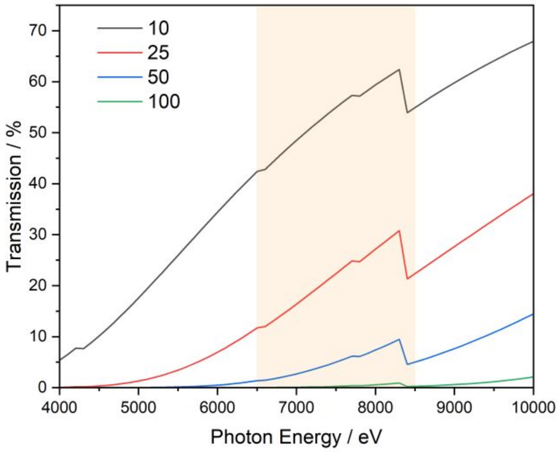

Figure S10: Calculation of the transmission with different penetration depths (micron) at Photon energy range from 4 keV to 10 keV containing the area of interest for Ni, Mn, and Co (6.5 keV to 8.5 keV – orange area in the plot). The depth in the calculation was meant to be double as the travelling pathway is double for the fluorescence measurement. The mixture of 57% NMC811 and 43% LICF was used to calculate the chemical formula of the cathode. The density is assumed to be 2.2 mg/cm^3 using the density of both materials with the corresponding percentage. The transmission through the whole 100 micron thickness of the cathode was not possible since the cathode contains Indium as a heavy element. Therefore, in this XAS measurement, the changes in transition metal valence are limited to material near cathode-current collector interface (up to 25 microns from this interface). The calculation was conducted using the online available calculation tool.[18]

References

(1) Alsaç, E. P.; Nelson, D. L.; Yoon, S. G.; Cavallaro, K. A.; Wang, C.; Sandoval, S. E.; Eze, U. D.; Jeong, W. J.; McDowell, M. T. Characterizing electrode materials and interfaces in solid-state batteries. *Chemical Reviews* **2025**, *125* (4), 2009–2119. DOI: 10.1021/acs.chemrev.4c00584.

(2) Doumeng, M.; Makhlouf, L.; Berthet, F.; Marsan, O.; Delbé, K.; Denape, J.; Chabert, F. A comparative study of the crystallinity of polyetheretherketone by using density, DSC, XRD, and Raman spectroscopy techniques. *Polymer Testing* **2021**, *93*, 106878. DOI: 10.1016/j.polymertesting.2020.106878.

(3) Taylor, T. P.; Ding, M.; Ehler, D. S.; Foreman, T. M.; Kaszuba, J. P.; Sauer, N. N. Beryllium in the environment: a review. *Journal of Environmental Science and Health, Part A* **2003**, *38* (2), 439–469. DOI: 10.1081/ESE-120016906.

(4) Dyadkin, V.; Pattison, P.; Dmitriev, V.; Chernyshov, D. A new multipurpose diffractometer PILATUS@ SNBL. *Synchrotron Radiation* **2016**, *23* (3), 825–829. DOI: 10.1107/S1600577516002411.

(5) Ashiotis, G.; Deschildre, A.; Nawaz, Z.; Wright, J. P.; Karkoulis, D.; Picca, F. E.; Kieffer, J. The fast azimuthal integration Python library: pyFAI. *Applied Crystallography* **2015**, *48* (2), 510–519. DOI: 10.1107/S1600576715004306.

(6) van Beek, W.; Safonova, O. V.; Wiker, G.; Emerich, H. SNBL, a dedicated beamline for combined in situ X-ray diffraction, X-ray absorption and Raman scattering experiments. *Phase Transitions* **2011**, *84* (8), 726–732. DOI: 10.1080/01411594.2010.549944.

(7) Ravel, B.; Newville, M. ATHENA, ARTEMIS, HEPHAESTUS: data analysis for X-ray absorption spectroscopy using IFEFFIT. *Synchrotron Radiation* **2005**, *12* (4), 537–541. DOI: 10.1107/S0909049505012719.

(8) Bianchini, M.; Roca-Ayats, M.; Hartmann, P.; Brezesinski, T.; Janek, J. There and back again—the journey of LiNiO2 as a cathode active material. *Angewandte Chemie International Edition* **2019**, *58* (31), 10434–10458. DOI: 10.1002/anie.201812472.

(9) Goonetilleke, D.; Schwarz, B.; Li, H.; Fauth, F.; Suard, E.; Mangold, S.; Indris, S.; Brezesinski, T.; Bianchini, M.; Weber, D. Stoichiometry matters: correlation between antisite defects, microstructure and magnetic behavior in the cathode material $Li_{1-z}Ni_{1+z}O_2$. *Journal of Materials Chemistry A* **2023**, *11* (25), 13468–13482. DOI: 10.1039/D3TA01621H.

(10) Li, J.; Liang, G.; Zheng, W.; Zhang, S.; Davey, K.; Pang, W. K.; Guo, Z. Addressing cation mixing in layered structured cathodes for lithium-ion batteries: A critical review. *Nano Materials Science* **2023**, *5* (4), 404–420. DOI: 10.1016/j.nanoms.2022.09.001.

(11) Liu, W.; Oh, P.; Liu, X.; Lee, M. J.; Cho, W.; Chae, S.; Kim, Y.; Cho, J. Nickel-rich layered lithium transition-metal oxide for high-energy lithium-ion batteries. *Angewandte Chemie International Edition* **2015**, *54* (15), 4440–4457. DOI: 10.1002/anie.201409262.

(12) Orlova, E. D.; Savina, A. A.; Abakumov, S. A.; Morozov, A. V.; Abakumov, A. M. Comprehensive study of Li^+/Ni^{2+} disorder in Ni-rich NMCs cathodes for Li-ion batteries. *Symmetry* **2021**, *13* (9), 1628. DOI: 10.3390/sym13091628.

(13) Zheng, J.; Ye, Y.; Liu, T.; Xiao, Y.; Wang, C.; Wang, F.; Pan, F. Ni/Li disordering in layered transition metal oxide: electrochemical impact, origin, and control. *Accounts of chemical research* **2019**, *52* (8), 2201–2209. DOI: 10.1021/acs.accounts.9b00033.

(14) Schlenker, R.; Hansen, A.-L.; Senyshyn, A.; Zinkevich, T.; Knapp, M.; Hupfer, T.; Ehrenberg, H.; Indris, S. Structure and diffusion pathways in Li_6PS_5Cl argyrodite from neutron diffraction, pair-distribution function analysis, and NMR. *Chemistry of materials* **2020**, *32* (19), 8420–8430. DOI: 10.1021/acs.chemmater.0c02418.

(15) Helm, B.; Schlem, R.; Wankmiller, B.; Banik, A.; Gautam, A.; Ruhl, J.; Li, C.; Hansen, M. R.; Zeier, W. G. Exploring aliovalent substitutions in the lithium halide superionic conductor $Li_{3-x}In_{1-x}Zr_xCl_6$ (0≤ x≤ 0.5). *Chemistry of materials* **2021**, *33* (12), 4773–4782. DOI: 10.1021/acs.chemmater.1c01348.

(16) Meindlhumer, M.; Brandt, L.; Zalesak, J.; Rosenthal, M.; Hruby, H.; Kopecek, J.; Salvati, E.; Mitterer, C.; Daniel, R.; Todt, J. Evolution of stress fields during crack growth and arrest in a brittle-ductile CrN-Cr clamped-cantilever analysed by X-ray nanodiffraction and modelling. *Materials & Design* **2021**, 198, 109365. DOI: 10.1016/j.matdes.2020.109365.

(17) Liu, J.; Lin, W.; Wang, Z.; Wang, Y.; Chen, T.; Zheng, J. Elastic Mechanics Study of Layered $Li(Ni_xMn_yCo_z)O_2$. *PRX Energy* **2024**, *3* (1), 013012. DOI: 10.1103/PRXEnergy.3.013012.

(18) Henke, B. L.; Gullikson, E. M.; Davis, J. C. X-ray interactions: photoabsorption, scattering, transmission, and reflection at E= 50-30,000 eV, Z= 1-92. *Atomic data and nuclear data tables* **1993**, *54* (2), 181–342. DOI: 10.1006/adnd.1993.1013.
